# Non-Alcoholic Steatohepatitis: Pathogenesis and Clinical Management

**DOI:** 10.1155/2015/153276

**Published:** 2015-07-28

**Authors:** Federico Salomone, Shira Zelber-Sagi, Fabio Galvano, Elisa Fabbrini

**Affiliations:** ^1^Division of Gastroenterology, Ospedale di Acireale, Azienda Sanitaria Provinciale di Catania, Catania, Italy; ^2^Liver Unit, Department of Gastroenterology, Tel-Aviv Medical Center, Tel-Aviv, Israel; ^3^School of Public Health, University of Haifa, Haifa, Israel; ^4^Department of Biomedical Sciences and Biotechnologies, University of Catania, Catania, Italy; ^5^Center for Human Nutrition and Atkins Center of Excellence in Obesity Medicine, Washington University School of Medicine, St. Louis, MO, USA

Nonalcoholic steatohepatitis (NASH) is emerging as the most prevalent liver disease in industrialized countries. NASH is associated with increased liver-related mortality due to cirrhotic and tumorigenic evolution and it is estimated that it will become the leading cause for liver transplantation in the next few years. Apart from liver morbidity, patients with NASH are also at increased risk for cardiovascular mortality, because these patients display endothelial dysfunction and a prothrombotic tendency. Thus, one hot topic in the field of NASH research is the identification of molecular factors involved in the progression of liver damage and cardiovascular dysfunction. One further challenge is to develop tools for the noninvasive diagnosis of NASH and for the assessment of fibrosis; although several efforts have been made, there is still a need to find more accurate and affordable tests. Finally, the main challenge for the clinician involved in the management of patients with NASH is treatment. Few patients are compliant with lifestyle modifications and pharmacological treatments, proposed so far, have been reported to have limited efficacy and safety.

This special issue on NASH reports original articles from basic and clinical researchers that give interesting contributions to the understanding of the pathogenesis and the clinical management of NASH. The issue contains also review articles from leading scientists that provide state-of-the-art view on specific topics. We hope that these articles may be of some inspiration to investigators and may help healthcare professionals in their clinical activity.

*Federico Salomone* *Shira Zelber-Sagi* *Fabio Galvano* *Elisa Fabbrini*

